# Predicting recurrence and recurrence-free survival in initially unresectable hepatocellular carcinoma: a novel nomogram for patients undergoing conversion hepatectomy with lenvatinib, PD-1 inhibitor, and interventional therapy

**DOI:** 10.3389/fimmu.2025.1602327

**Published:** 2025-07-30

**Authors:** Cheng Xu, Zhihong Tang, Meng Wei, Danxi Liu, Qingqing Pang, Baishan Huang, Xinglin Mo, Feixiang Wu

**Affiliations:** ^1^ Department of Hepatobiliary Surgery, Guangxi Medical University Cancer Hospital, Nanning, China; ^2^ Key Laboratory of Early Prevention and Treatment for Regional High Frequency Tumor (Guangxi Medical University), Ministry of Education, Nanning, China

**Keywords:** hepatocellular carcinoma, hepatectomy, recurrence, recurrence-free survival, nomogram model

## Abstract

**Purpose:**

This research aims to develop prognostic nomograms to predict tumor recurrence and recurrence-free survival (RFS) in individuals with initially unresectable hepatocellular carcinoma (uHCC) who were later subjected to conversion hepatectomy following lenvatinib, PD-1 inhibitors, and interventional (LPI) therapy.

**Methods:**

We performed a retrospective review of clinical information from 150 individuals diagnosed with HCC who underwent conversion hepatectomy following LPI therapy between November 2019 and December 2024. Independent predictors linked to recurrence and RFS were identified through comprehensive univariate and multivariate analyses, and the identified factors were subsequently integrated into nomogram models. Receiver operating characteristic (ROC) curves, calibration plots, and the concordance index (C-index) were employed to evaluate the predictive performance of the nomograms.

**Results:**

Our investigation identified several key risk factors for recurrence, including age, tumor number, tumor differentiation, preoperative prognostic nutritional index (PNI), preoperative systemic immune-inflammation index (SII), and postoperative protein induced by vitamin K absence or antagonist-II (PIVKA-II) level. For RFS, significant predictors included tumor number, tumor differentiation, preoperative SII, postoperative PIVKA-II, and postoperative alpha-fetoprotein (AFP) levels. The nomograms exhibited strong predictive performance, achieving a C-index of 0.837 (95% CI: 0.775–0.896) for recurrence prediction and 0.837 (95% CI: 0.788–0.886) for RFS. Our nomogram for recurrence prediction outperformed traditional staging systems like China Liver Cancer (CNLC) staging and Barcelona Clinic Liver Cancer (BCLC). Calibration curves and discriminative ability assessments confirmed the nomograms’ reliability in predicting actual outcomes and stratifying patients into distinct prognostic subgroups with significant RFS differences across risk categories.

**Conclusions:**

The nomogram models established in this research provide an exceptionally accurate and individualized method for predicting recurrence and RFS in initially uHCC patients undergoing LPI-based conversion hepatectomy, potentially aiding clinicians in devising tailored treatment plans and enhancing patient outcomes.

## Introduction

Globally, hepatocellular carcinoma (HCC) is a highly prevalent malignancy with a significant impact on cancer-related mortality (1). Radical hepatectomy is the preferred treatment for early HCC (2). Nevertheless, a considerable number of individuals are identified with advanced-stage diseases that are not amenable to surgical removal. This clinical challenge has spurred interest in multimodal therapeutic strategies for initially unresectable HCC (uHCC). The integration of systemic and interventional approaches has demonstrated substantial potential in enhancing outcomes for patients with uHCC (3).

In the management of advanced HCC, lenvatinib is widely endorsed as a systemic treatment option, achieving satisfactory survival times and therapeutic efficacy (4). Therapeutic strategies targeting the PD-1 and PD-L1 immune checkpoint pathways have been demonstrated to augment T-cell activity and demonstrate significant tumor-suppressing effects by augmenting T-cell activity, demonstrating favourable clinical benefits in advanced HCC patients (5, 6). Recent clinical studies have highlighted the synergistic benefits of combining lenvatinib with anti-PD-1 antibodies, which not only maintains a manageable safety profile while enhancing efficacy. This combination therapy has emerged as a promising approach for initially uHCC, potentially offering greater translational potential compared to monotherapy in achieving radical surgical resection (7). While systemic therapies have shown significant promise, local therapeutic interventions also play a crucial role in enhancing therapeutic outcomes for advanced-stage HCC patients. Transarterial chemoembolization (TACE) has been shown to effectively enhance tumor response in uHCC patients, leading to better survival outcomes (8, 9). Additionally, hepatic arterial infusion chemotherapy (HAIC) has been demonstrated to effectively elevate local hepatic drug levels while minimizing systemic toxicity. These findings indicate that HAIC may markedly enhance overall survival (OS) in advanced HCC patients, establishing itself as a promising and safe treatment alternative for those with unresectable large HCC (10).

By integrating systemic and local therapeutic approaches, comprehensive treatment strategies can be developed to maximize therapeutic benefits. A comprehensive meta-analysis has highlighted the effectiveness of the integrated treatment approach involving lenvatinib, PD-1 inhibitors, and interventional therapy (LPI) in offering enhanced therapeutic benefits (11). In a separate retrospective study, the HAIC of the FOLFOX regimen combined with lenvatinib and anti-PD-1 antibodies was found to be a successful primary therapy for HCC with portal vein tumor thrombus with tolerable adverse reactions (12).

In conclusion, tumor downstaging through various therapeutic interventions is a promising research direction for transforming initially uHCC cases into those suitable for radical resection. Conversion hepatectomy with the LPI regimen enables surgical resection of initially uHCC and has demonstrated superior survival benefits compared to PD-1 inhibitors, TACE, or their combination, emerging as a pivotal therapeutic strategy (13, 14). Despite significant progress in HCC treatment, patients undergoing conversion hepatectomy continue to face a substantial risk of tumor recurrence (15, 16). Achieving long-term survival remains a formidable challenge due to the high postoperative recurrence rate. Research on recurrence after conversion hepatectomy and the impact of prior LPI regimen in HCC patients is still limited. Considering the heightened risk of recurrence after hepatectomy, promptly detecting recurrence and identifying patients suitable for adjuvant or neoadjuvant therapies are of utmost significance. Precise post-hepatectomy prognostic information is essential for guiding clinical decisions and managing patient expectations, as it can assist physicians in determining appropriate adjuvant therapies and the best follow-up frequency. Moreover, the use of postoperative combination therapies may enhance the prognosis for individuals prone to tumor recurrence.

Currently, there is a lack of precise and readily applicable predicting instruments. Therefore, it is crucial to develop a framework that consolidates relevant factors from perioperative clinical and pathological data. This study aims to retrospectively evaluate patients with HCC who received conversion hepatectomy following an LPI regimen. The objective is to establish an accessible nomogram-based prediction model to estimate the postoperative recurrence risk and recurrence-free survival (RFS). By offering personalized and highly accurate risk assessments, this model can function as a valuable instrument in guiding clinical management strategies for HCC patients.

## Materials and methods

### Patients

This investigation retrospectively reviewed the medical records of 150 consecutive individuals with HCC who received conversion therapy with an LPI regimen as their initial treatment, followed by hepatectomy at Guangxi Medical University Cancer Hospital from November 2019 to December 2024. The tumors were classified as initially unresectable based on the following criteria: inadequate future liver remnant volume resulting from extensive tumor burden, the presence of multiple lesions, or vascular invasion, all of which prevented safe R0 resection.

Patients were eligible for conversion hepatectomy if they fulfilled the specified criteria: *a*) achievement of disease stabilization following conversion therapy; *b*) LPI regimen as initial treatment; *c*) complete (R0) tumor resection; *d*) liver function classified as Child-Pugh A or B; and *e*) an Eastern Cooperative Oncology Group (ECOG) performance score of 2 or lower prior to treatment. The Chinese guidelines specify that the inclusion criteria for surgery require an ECOG of 2 or lower or a Child-Pugh classification of A or B. Patients with an ECOG score greater than 3 or a Child-Pugh classification of C are ineligible for surgery due to poor liver function and overall health (17). Individuals were excluded if they presented with: *a*) severe dysfunction of the heart, lungs, or kidneys prior to conversion therapy or hepatectomy; *b*) the presence of distant metastasis detected during the initial follow-up; *c*) incomplete clinicopathological data or lack of follow-up information; *d*) a history of other malignancies; or *e*) tumor progression during conversion therapy. The study protocol underwent review and received approval from our Ethics Committee at Guangxi Medical University Cancer Hospital (approval number: LW2025014). Per the Declaration of Helsinki and given the retrospective design of the study with minimal risk to participants, the Institutional Review Board granted a waiver for informed consent.

Clinical and pathological data were gathered at three critical time points: *a*) before the initiation of the first cycle of LPI, *b*) during the period between the last cycle of LPI and the subsequent hepatectomy, and *c*) at the initial follow-up visit following hepatectomy. These data included comprehensive assessments of tumor characteristics, liver function, treatment responses, and any adverse events, ensuring a thorough evaluation of the individual’s clinical progression throughout the entire therapeutic journey.

### Conversion therapy and evaluation

The LPI combination therapy was administered as previously detailed (18). In summary, all participants underwent TACE or HAIC before commencing combination therapy with lenvatinib and PD-1 inhibitors. When abdominal-enhanced computed tomography (CT) or contrast-enhanced magnetic resonance imaging (MRI) revealed significant vascularity around the tumor, TACE was conducted using an appropriate volume of iodized oil combined with a lobaplatin or epirubicin emulsion. For HAIC, a modified FOLFOX regimen was utilized, consisting of oxaliplatin at 85 mg/m², leucovorin at 400 mg/m², and a fluorouracil bolus of 400 mg/m² on Day 1, followed by a 46-hour infusion of fluorouracil at 2400 mg/m². The treatment cycle was repeated every 3 weeks. Patients initiated lenvatinib and PD-1 inhibitor therapy no later than 7 days after TACE or HAIC. Lenvatinib was dosed at 12 mg daily for patients ≥60 kg and 8 mg daily for those <60 kg. PD-1 inhibitors (sintilimab, tislelizumab, or camrelizumab, all at 200 mg) were delivered via intravenous infusion on a tri-weekly schedule alongside lenvatinib. Therapy was discontinued for disease progression, intolerable adverse events, or successful conversion to resectability.

Tumor response was assessed according to the modified Response Evaluation Criteria in Solid Tumors (mRECIST) guidelines (19) by two board-certified hepatobiliary surgeons (CX and MW), who assessed treatment efficacy and recurrence via comparative analysis of target lesion changes on pre-/post-intervention imaging (enhanced CT and enhanced MRI). Follow-up evaluations included laboratory tests such as routine blood tests, liver function assessments, and tumor marker evaluations. For liver function assessments, we collected data on alanine aminotransferase (ALT), aspartate aminotransferase (AST), total bilirubin (TBIL), albumin (ALB), and prothrombin time (PT). These indicators were chosen based on their established roles in evaluating liver function. The tumor marker evaluations encompassed measurements of alpha-fetoprotein (AFP), PIVKA-II, and carbohydrate antigen 19-9 (CA 19-9), which are commonly recognized markers for hepatocellular carcinoma. Additionally, we evaluated various inflammatory indicators, including the prognostic nutritional index (PNI), the systemic immune-inflammation index (SII), the systemic inflammatory response index (SIRI), the neutrophil-to-lymphocyte ratio (NLR), and the platelet-to-lymphocyte ratio (PLR). These indices have demonstrated prognostic significance in hepatocellular carcinoma (20).

### Surgical treatment and follow-up

When the conversion therapy met the resectability criteria—specifically, sufficient residual liver volume, R0 resection margins, and no contraindications for hepatectomy—then hepatectomy could be performed. Preoperative evaluations were mandatory before hepatectomy and included tests for hepatic and renal function, coagulation function, and the measurement of liver cancer-specific biomarkers. These assessments also covered the ECOG performance score, abdominal-enhanced CT, cardiopulmonary function testing, and measurement of tumor markers, including AFP, PIVKA-II levels.

Postoperative follow-up after resection comprised comprehensive assessments, including standard hematological profiling, serum tumor marker analysis, hepatic and coagulation function tests, and abdominal-enhanced CT scans. These evaluations were performed monthly or bimonthly during the first year and quarterly thereafter. For statistical analysis, we used data from the first follow-up after hepatectomy, in line with our initial data collection framework. Tumor recurrence was defined by two criteria: *a*) the emergence of newly detected lesions with typical HCC features in two consecutive radiological investigations, and *b*) no hepatic extra-metastases prior to hepatectomy. The primary endpoints assessed in this investigation were tumor recurrence and RFS, with RFS calculated as the time from surgical resection to HCC recurrence or the last follow-up (censored on December 31, 2024).

### Statistical analysis

Quantitative variables were presented as mean ± standard deviation or median (interquartile range), while qualitative variables were described using frequency and percentage. Comparisons of quantitative variables were performed using the t-test or Wilcoxon rank-sum test, and comparisons of qualitative variables were conducted using the chi-square test or Fisher’s exact test. Optimal cutoff values for age and inflammatory biomarkers were determined using the maximally selected rank statistic method implemented in the “surv_cutpoint” function from the “survminer” R package, stratified by overall survival (OS) outcomes (21, 22). Univariate analysis was initially performed to identify potential predictors of tumor recurrence and RFS. Variables with *P* < 0.2 in univariate analysis were retained for multivariable logistic regression (for tumor recurrence) and Cox proportional hazards regression (for RFS). Factors achieving statistical significance (*P* < 0.05) in multivariable models were integrated into a nomogram. The predictive model underwent rigorous internal validation using 1000 bootstrap resamples to adjust for overfitting. Model performance was evaluated by the concordance index (C-index) for discrimination and calibration curves with the Hosmer-Lemeshow test for accuracy. Additionally, the area under the receiver operating characteristic (AUROC) curve was calculated to assess predictive probability. Statistical analyses and graphical presentations were performed utilizing R software (version 4.3.3), with nomogram development and validation performed using the “rms” and “survminer” packages.

## Results

### Patient characteristics

In this retrospective analysis, we analyzed data from 150 consecutive individuals with initially uHCC who received the LPI regimen as first-line conversion therapy before undergoing hepatectomy. Among the enrolled patients, 67.3% (101/150) were treated with camrelizumab, 10.0% (15/150) with sintilimab, and 22.7% (34/150) with tislelizumab. For interventional therapy, 81.3% (122/150) underwent TACE, while 18.7% (28/150) received HAIC.

The median follow-up period was 14.5 months, ranging from 1 to 56.4 months. During this period, tumor recurrence was detected in 71 patients (47.3%). The median RFS was 9.58 months, with a 95% confidence interval of 5.68 to 13.96 months. The RFS rates at 1, 2, and 3 years were 52%, 22%, and 6.7%, respectively. Comprehensive clinicopathological features of the study cohort are detailed in **Table 1**.

**Table 1:** Participant characteristics.

Variable	Number of patients (%)
Age, years, median (IQR)	50(44-58)
Age (≤ 45/> 45years)	51(34)/99(66)
Gender (male/female)	130(86.7)/20(13.3)
Tumor size (≥ 5cm/< 5cm)	139(92.6)/11(7.4)
Tumor number(solitary/multiple)	90(60)/60(40)
MVI (no/yes)	97(64.6)/53(35.4)
BCLC stage(A/B/C)	43(28.6)/48(32)/59(39.4)
CNLC stage (I/II/III)	43(28.6)/37(24.6)/70(47.7)
ECOG PS (0/1/2)	102(68)/39(26)/9(6)
Differentiation (well/moderate/poor)	65(43.3)/34(22.6)/51(34.1)
Pre-LPI serum tests	
Platelets (< 100/ ≥ 100) (× 10^3^/μl)	2(1.3)/148(98.7)
PT (≤ 12/ > 12)	55(36.6)/95(63.3)
AFP (< 400/ ≥ 400) (ng/ml)	103(68.6)/47(31.3)
PIVKA-II (< 40/ ≥ 40) (mAU/mL)	85(56.6)/65(43.3)
CA19-9 (≤ 35/ > 35) (U/ml)	104(69.3)/46(30.7)
ALB (<35/≥35) (g/l)	33(22)/117(78)
TBIL (≤ 20/ > 20) (μmol/l)	118(78.6)/32(21.3)
Preoperative serum tests	
Platelets (< 100/ ≥ 100) (× 10^3^/μl)	14(9.3)/136(90.7)
PT (≤ 12/ > 12)	96(64)/54(36)
AFP (< 400/ ≥ 400) (ng/ml)	129(86)/21(14)
PIVKA-II (< 40/ ≥ 40) (mAU/mL)	103(68.6)/47(31.4)
CA19-9 (≤ 35/ > 35) (U/ml)	139(92.6)/11(7.4)
ALB (<35/≥35) (g/l)	56(37.3)/94(62.7)
TBIL (≤ 20/ > 20) (μmol/l)	140(93.3)/10(6.7)
Postoperative serum tests	
Platelets (< 100/ ≥ 100) (× 10^3^/μl)	11(7.3)/139(92.7)
PT (≤ 12/ > 12)	128(85.3)/22(14.7)
AFP (≤ 25/ 25-400/ ≥ 400) (ng/ml)	47(31.3)/93(62)/10(6.7)
PIVKA-II (< 40/ ≥ 40) (mAU/mL)	119(79.3)/31(20.7)
CA19-9 (≤ 35/ > 35) (U/ml)	120(80)/30(20)
ALB (<35/≥35) (g/l)	23(15.3)/127(84.7)
TBIL (≤ 20/ > 20) (μmol/l)	127(84.7)/23(15.3)
Pre-LPI inflammation index	
Pre-LPI PNI (< 45/≥ 45)	56(37.3)/94(62.7)
Pre-LPI NLR (≥1.8/<1.8)	95(63.3)/55(37.7)
Pre-LPI SII (≥322/<322)	105(70)/45(30)
Pre-LPI SIRI (≥1.4/<1.4)	64(42.7)/86(57.3)
Pre-LPI PLR (≥92/<92)	102(68)/48(32)
Preoperative inflammation index	
Preoperative PNI (< 45/≥ 45)	100(66.7)/50(33.3)
Preoperative NLR (≥1.8/<1.8)	88(54.7)/62(41.3)
Preoperative SII (≥322/<322)	85(56.7)/65(43.3)
Preoperative SIRI (≥1.4/<1.4)	56(37.3)/94(62.7)
Preoperative PLR (≥92/<92)	108(70)/42(30)

IQR, interquartile range; MVI, Microvascular Invasion; BCLC, Barcelona‐Clinic Liver Cancer; CNLC, China Liver Cancer Staging; ECOG PS, Eastern Cooperative Oncology Group performance score; LPI, lenvatinib, PD-1 inhibitors, and interventional therapy; AFP, alpha‐fetoprotein; PIVKA-II, protein induced by vitamin K absence or antagonist-II; CA19-9, carbohydrate antigen 19-9; ALB, albumin; TBIL, total bilirubin; PT, prothrombin time; PNI, Prognostic nutritional index; NLR, Neutrophil to lymphocyte ratio; SII, Systemic immune-inflammation index; SIRI, systemic inflammatory response index; PLR, Platelet to lymphocyte ratio.

### Independent predictors of recurrence and RFS

Multivariate logistic regression analysis identified that age [odds ratio (OR) = 4.563; 95% confidence interval (CI) = 1.543–14.904], tumor number (OR = 4.802; 95% CI = 1.767–14.478), tumor differentiation (OR=2.243; 95% CI = 1.238–4.241), preoperative prognostic nutritional index (PNI) (OR = 7.296; 95% CI = 2.119–29.207), preoperative SII (OR = 7.477; 95% CI = 1.675–39.491) and postoperative PIVKA-II level (OR = 7.393; 95% CI = 1.843–37.549) were recognized as independent predictors of tumor recurrence (**Table 2**).

**Table 2:** Univariate and multivariate logistic regression analyses of prognostic factors for recurrence probability.

Variables	Univariate analysis	P	Multivariate analysis	P
	OR (95% CI)		OR (95% CI)	
Age (≤ 45/> 45years)	2.021 (1.023–4.054)	0.044	4.563 (1.543-14.904)	0.008
BCLC stage				
B vs. A	1.497 (0.752–3.001)	0.251		
C vs. A	1.580 (0.819–3.072)	0.173		
Tumor number (multiple vs. solitary)	2.983 (1.528–5.950)	0.001	4.802 (1.767-14.478)	0.003
Differentiation	2.617 (1.758-3.995)	< 0.001	2.243 (1.238-4.241)	0.009
Pre-LPI monocyte (×10^9^/L)	3.703 (0.782-19.204)	0.106		
Pre-LPI ALT (> 50 vs. ≤ 50) (U/L)	1.768 (0.844-3.770)	0.134		
Pre-LPI PT (> 12 vs. ≤ 12)	1.598 (0.819-3.159)	0.172		
Pre-LPI PIVKA-II (≥ 40 vs.< 40) (mAU/mL)	1.588 (0.831-3.059)	0.163		
Pre-LPI NLR (≥1.8 vs. <1.8)	2.028 (1.034-4.058)	0.042		
Pre-LPI PLR (≥92 vs.<92)	2.678 (1.316-5.643)	0.008		
Pre-LPI SII (≥322 vs.<322)	2.286 (1.117-4.832)	0.026		
Pre-LPI SIRI (≥1.4 vs.<1.4)	2.347 (1.219-4.591)	0.011		
Preoperative AFP (≥ 400 vs. < 400) (ng/ml)	2.526 (0.983-7.053)	0.062		
Preoperative PNI (< 45 vs. ≥ 45)	2.005 (1.006-4.087)	0.051	7.296 (2.119-29.207)	0.002
Preoperative NLR (≥1.8 vs. <1.8)	1.815 (0.942-3.544)	0.077		
Preoperative SII (≥322 vs.<322)	2.993 (1.538-5.965)	0.001	7.477 (1.675-39.491)	0.012
Preoperative SIRI (≥1.4 vs.<1.4)	2.381 (1.218-4.738)	0.012		
Postoperative PIVKA-II (≥ 40 vs.< 40) (mAU/mL)	5.252 (2.192-14.093)	< 0.001	7.393 (1.843-37.549)	0.008
Postoperative AFP (ng/ml)				
25-400 vs. ≤ 25	1.577 (0.812-3.098)	0.181		
≥ 400 vs. ≤ 25	3.913 (2.055-7.989)	< 0.001		

BCLC, Barcelona‐Clinic Liver Cancer; LPI, lenvatinib, PD-1 inhibitors, and interventional therapy; ALT, Alanine aminotransferase; PT, prothrombin time; PIVKA-II, protein induced by vitamin K absence or antagonist-II; AFP, alpha‐fetoprotein; PNI, Prognostic nutritional index; NLR, Neutrophil to lymphocyte ratio; SII, Systemic immune-inflammation index; SIRI, systemic inflammatory response index; PLR, Platelet to lymphocyte ratio.

Furthermore, Multivariate Cox regression analysis pinpointed five key predictors associated with RFS. These factors incorporated tumor differentiation (HR = 1.544; 95% CI = 1.072-2.226), preoperative SII (HR = 3.918; 95% CI = 1.586-9.683), postoperative PIVKA-II level (HR = 4.954; 95% CI = 2.582-9.505), tumor number (HR = 2.7808; 95% CI = 1.577-4.903), and postoperative AFP levels (25–400 ng/mL vs. ≤25 ng/mL: HR = 2.591; 95% CI = 1.153-5.823; ≥400 ng/mL vs. ≤25 ng/mL: HR = 44.793; 95% CI = 13.145-152.633). A comprehensive overview of these RFS predictors is presented in **Table 3**.

**Table 3:** Univariate and multivariate Cox regression analyses of prognostic factors for recurrence-free survival.

Variables	Univariate analysis	P	Multivariate analysis	P
	HR (95% CI)		HR (95% CI)	
Age (≤ 45/> 45years)	1.89 (1.178–3.033)	0.008		
BCLC stage (B vs. A)	1.558 (0.951-2.553)	0.078		
MVI (1 vs. 0)	1.51 (0.9138-2.495)	0.108		
Tumor number (multiple vs. solitary)	2.029 (1.272-3.236)	0.003	2.781 (1.577-4.903)	< 0.001
Differentiation	2.158 (1.616-2.884)	< 0.001	1.544 (1.072-2.226)	0.02
Pre-LPI Neutrophil (×10^9^/L)	1.091 (0.9864-1.206)	0.091		
Pre-LPI monocyte (×10^9^/L)	2.456 (0.8563-7.047)	0.095		
Pre-LPI PIVKA-II (> 40 vs. ≤ 40) (mAU/mL)	1.51 (0.9463-2.411)	0.084		
Pre-LPI NLR (≥1.8 vs. <1.8)	1.964 (1.171-3.295)	0.011		
Pre-LPI PLR (≥92 vs.<92)	2.067 (1.165-3.667)	0.013		
Pre-LPI SII (≥322 vs.<322)	2.018 (1.14-3.57)	0.016		
Pre-LPI SIRI (≥1.4 vs.<1.4)	2.081 (1.3-3.331)	0.002		
Preoperative AFP (≥ 400 vs. < 400) (ng/ml)	2.439 (1.347-4.415)	0.003		
Preoperative NLR (≥1.8 vs. <1.8)	1.606 (0.982- 2.626)	0.059		
Preoperative SII (≥322 vs.<322)	2.402 (1.431-4.033)	< 0.001	3.918 (1.586-9.683)	0.003
Preoperative SIRI (≥1.4 vs.<1.4)	1.721 (1.082-2.74)	0.022		
Postoperative PIVKA-II (> 40 vs. ≤ 40) (mAU/mL)	4.053 (2.447-6.71)	< 0.001	4.954 (2.582-9.505)	< 0.001
Postoperative AFP (ng/ml)				
25-400 vs. ≤ 25	2.147 (1.273-3.622)	0.004	2.591 (1.153-5.823)	0.021
≥ 400 vs. ≤ 25	24.65 (10.23-59.4)	< 0.001	44.793 (13.145-152.633)	< 0.001

BCLC, Barcelona‐Clinic Liver Cancer; MVI, Microvascular Invasion; LPI, lenvatinib, PD-1 inhibitors, and interventional therapy; PIVKA-II, protein induced by vitamin K absence or antagonist-II; AFP, alpha‐fetoprotein; NLR, Neutrophil to lymphocyte ratio; SII, Systemic immune-inflammation index; SIRI, systemic inflammatory response index; PLR, Platelet to lymphocyte ratio.

### Construction of the prognostic nomograms

**Figure 1:**
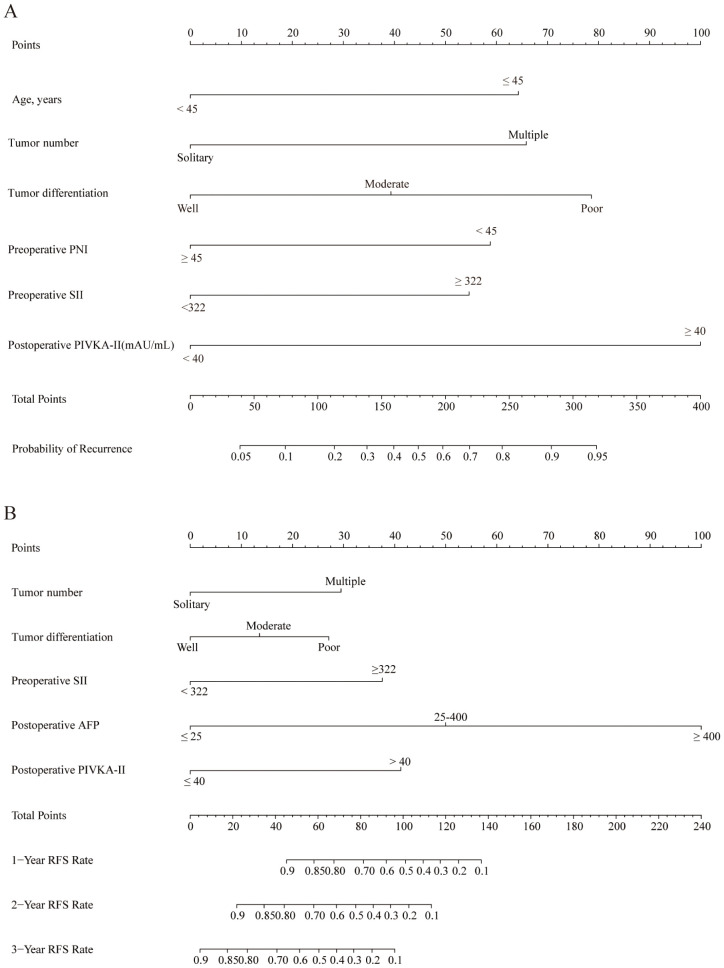
Nomograms to predict recurrence and RFS of patients with initially uHCC who underwent conversion liver resection and previously received LPI regimen. **(A)** The nomogram to predict the probability of tumor recurrence was generated based on 6 independent prognostic factors. **(B)** The nomogram to predict the RFS was generated based on 5 independent prognostic factors. uHCC, unresectable hepatocellular carcinoma; RFS, recurrence-free survival; LPI, lenvatinib, PD-1 inhibitors, and interventional therapy.

**Figures 1A, B** present nomograms developed to predict the probability of tumor recurrence and RFS among individuals with initially uHCC who underwent conversion liver resection following the LPI regimen. The recurrence nomogram was constructed using prognostic factors identified through multivariate analysis, including age, tumor number, tumor differentiation, preoperative PNI, preoperative SII, and postoperative PIVKA-II level. The RFS nomogram was constructed based on five key predictive factors: tumor number, tumor differentiation, preoperative SII, postoperative PIVKA-II level, and postoperative AFP level.

Higher scores on these nomograms are associated with an increased likelihood of tumor recurrence or reduced RFS. For instance, a 60-year-old patient with a solitary tumor, poorly differentiated tumor cells, preoperative PNI < 45, preoperative SII > 322, and postoperative PIVKA-II ≥ 40 mAU/mL would receive a total score of 239 points. This score breaks down as follows: 78.5 points allocated to tumor differentiation, 58.5 points attributed to preoperative PNI, 55 points designated for preoperative SII, and 100 points assigned to postoperative PIVKA-II, with no points assigned for age or tumor number. The predicted probability of recurrence for this patient is 70%.

Similarly, a patient with poorly differentiated tumors, multiple tumors, preoperative SII ≤ 322, postoperative AFP ≤ 25 ng/mL, and postoperative PIVKA-II ≥ 40 mAU/mL would accumulate a total score of 97.5 points. This score is composed of 29.5 points for tumor number, 27 points for tumor differentiation, and 41 points for postoperative PIVKA-II, with no points assigned for preoperative SII or postoperative AFP. The projected RFS rates for this individual are 55.0%, 27.0%, and 1.0% at 1, 2, and 3 years, respectively.

### Efficacy of the nomogram

**Figure 2:**
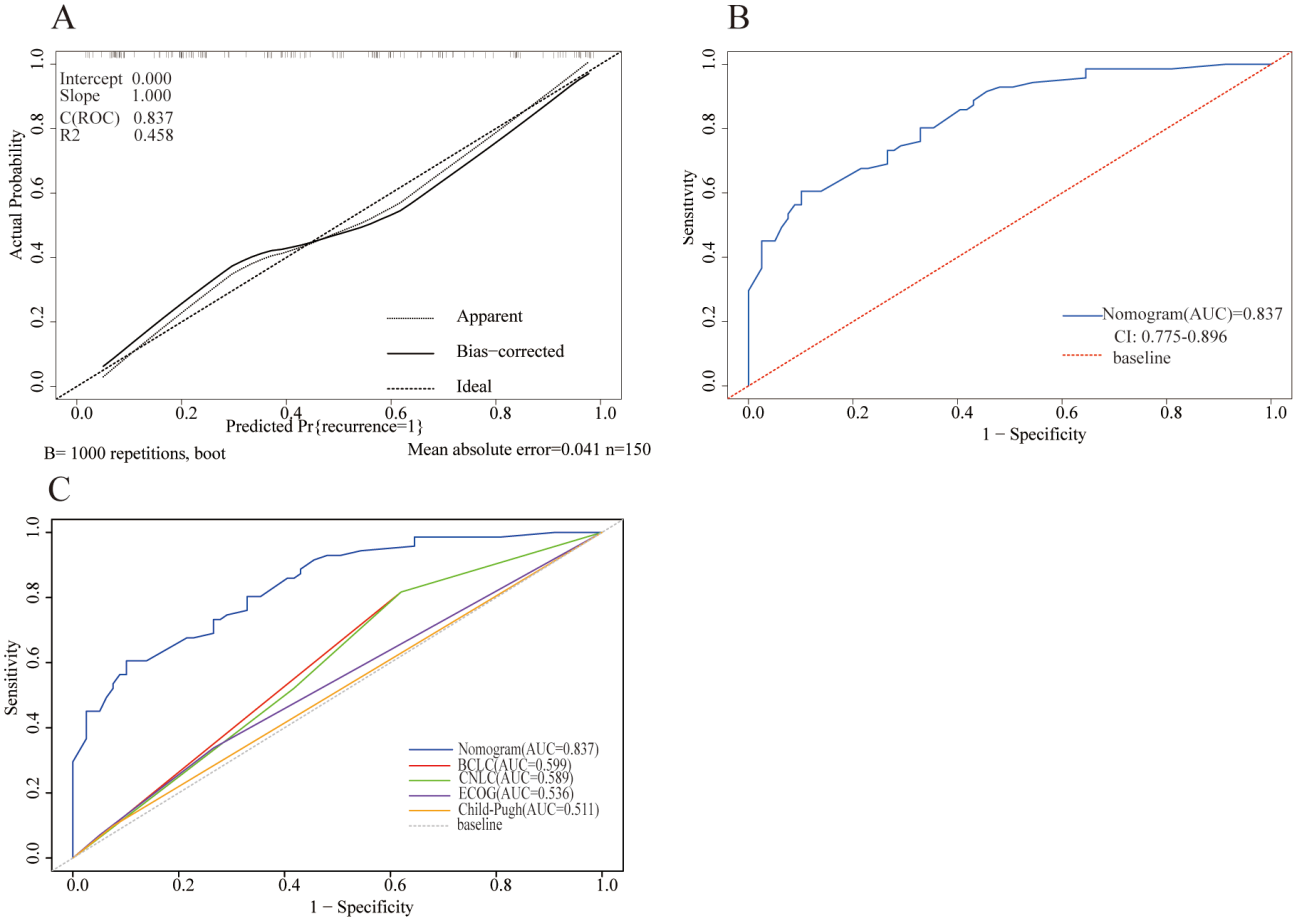
Model performance of the nomogram for predicting tumor recurrence. **(A)** Calibration plot comparing predicted and actual probability of recurrence. **(B)** Receiver operator characteristic (ROC) curve and the corresponding area of the predictive model for the probability of recurrence. **(C)** ROC curves showing the performance of the nomogram and other models for predicting tumor recurrence in the cohort.

For internal validation, the bootstrap validation approach was employed. The nomograms exhibited exceptional precision in predicting the probability of tumor recurrence, with an initial C-index of 0.837 and a bootstrap-adjusted C-index of 0.811. Additionally, the calibration curve showed strong alignment between predicted results and actual observations, reflected by a Brier score of 0.163 (**Figure 2A**). The AUROC curve for predicting tumor recurrence was 0.837 (95% CI: 0.775–0.896) (**Figure 2B**).

Compared with existing staging systems, the nomogram showed superior performance in predicting tumor recurrence probability. It achieved an AUROC value of 0.837, outperforming the Barcelona Clinic Liver Cancer (BCLC) staging system (0.599), the China Liver Cancer (CNLC) staging system (0.589), the ECOG performance score (0.536), and the Child-Pugh classification (0.511) (**Figure 2C**).

**Figure 3:**
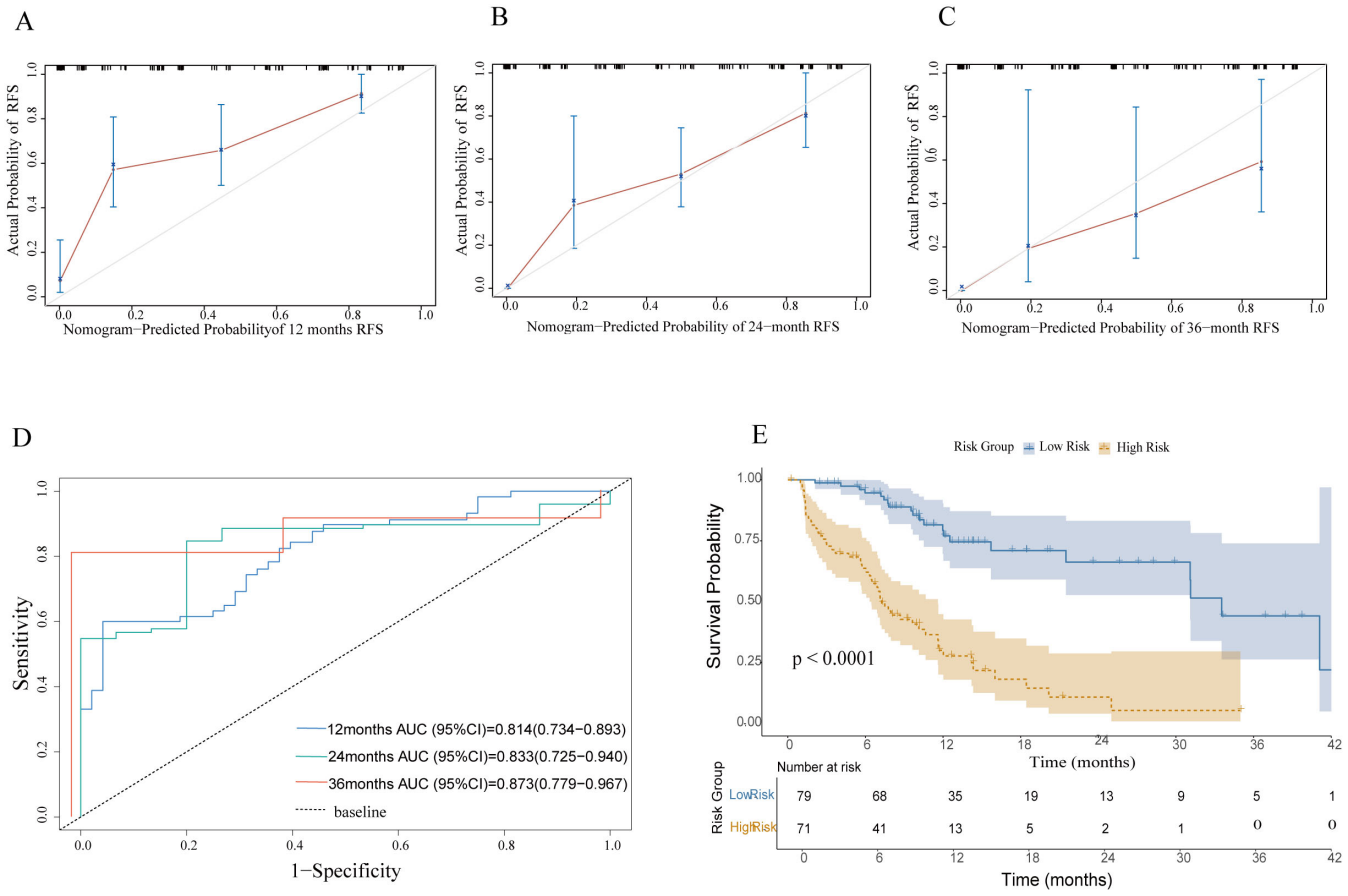
Model performance. **(A–C)** Calibration plots for predicting 1-year,2-year, and 3-year RFS. **(D)** ROC curves evaluating the nomogram's predictive performance for 12, 24, and 36 months in the cohort. **(E)** RFS was compared between patients with nomogram low-risk and high-risk scores in the cohort. RFS, recurrence-free survival.

Regarding the prediction of RFS, the nomogram achieved a C-index of 0.837 (95% CI: 0.788–0.886). The calibration plot demonstrated that the nomogram-prediction RFS possibilities at 1, 2, and 3 years closely aligned with the actual observed RFS probabilities in the patient cohort (**Figures 3A–C**). The AUROC curves for 12-, 24-, and 36-month RFS were 0.814 (95% CI: 0.734–0.893), 0.833 (95% CI: 0.725–0.940), and 0.873 (95% CI: 0.779–0.967), respectively (**Figure 3D**).

In this study, we utilized a nomogram-generated risk score to stratify patients into low-risk and high-risk groups based on the median score calculated by the nomogram. This approach quantifies risk scores to analyze patient prognosis. We employed the Kaplan-Meier survival analysis method to systematically compare the RFS between the two groups. RFS is a key metric for evaluating tumor treatment efficacy and predicting patient outcomes. Our survival curve analysis revealed that, across the entire study cohort, the high-risk group exhibited markedly lower RFS compared to the low-risk group. This difference was evidenced by a higher incidence of recurrence or mortality events in the high-risk patients (P < 0.0001). These findings show that the nomogram-based risk-scoring system can effectively distinguish patient groups with different risk levels and provide a reliable basis for prognostic evaluation (**Figure 3E**).

## Discussion

Hepatic resection is the gold standard for treating HCC with curative intent, offering the possibility of long-term survival in selected patients. However, many are ineligible for this crucial procedure due to strict criteria and technical challenges. Research shows that nearly 70% of HCC cases encounter barriers like locally advanced disease or metastatic spread (17). As a result, these patients face limited treatment options and poor outcomes, with 5-year survival rates below 10% for those unable to undergo surgery (23). Consequently, the rates of resection need to be increased without delay. Recent advancements in conversion therapies have broadened the surgical options for initially inoperable tumors, thus enhancing survival outcomes (24). Nevertheless, the high rate of postoperative recurrence especially in patients undergoing conversion therapy, continues to pose a major obstacle to achieving long-term survival. Therefore, accurately predicting recurrence risk and implementing preventive strategies are crucial to enhance patient outcomes.

Our study documented postoperative tumor recurrence in 47.3% (71/150) of LPI regimen-based conversion hepatectomy patients. Several high-risk predictors for tumor recurrence and RFS were identified, including age, tumor number, tumor differentiation, preoperative PNI, preoperative SII, postoperative AFP level, and postoperative PIVKA-II level. We developed two prognostic nomograms integrating clinicopathological parameters to more accurately assess recurrence potential and RFS following LPI regimen-based conversion hepatectomy. These nomograms are predictive models created to specifically assess the recurrence and RFS in patients with HCC undergoing conversion hepatectomy based on the LPI regimen. Our study complements existing research by focusing on the critical aspect of postoperative recurrence, which significantly impacts long-term outcomes for this patient population. This tool can identify patients at high risk of tumor recurrence and compromised RFS, thereby guiding personalized postoperative treatment strategies and optimal follow-up intervals. The nomograms demonstrated robust predictive accuracy with a C-index of 0.837 for both recurrence and RFS prediction. Furthermore, calibration curves showed high concordance between predicted and observed outcomes.

A significant advantage of this study is the inclusion of key variables associated with prognosis after hepatectomy, as reported in prior studies. For instance, AFP, a crucial HCC tumor marker, is incorporated into many liver cancer prognostic models (25). In recent years, PIVKA-II has also been utilized as an HCC tumor marker, complementing AFP to enhance HCC diagnosis and management (26, 27). Thus, Serum tumor marker tests are commonly used in postoperative follow-up to detect tumor recurrence as early as possible. Recent studies have shown that multiple tumor lesions, necessitating prompt intervention due to their strong association with HCC recurrence (28), and poorly differentiated tumors, which exhibit greater biological aggressiveness and may significantly impact recurrence and long-term survival (29), represent critical determinants requiring stratified management in HCC treatment. Our findings are consistent with prior research indicating that recurrence is closely associated with key clinical and histopathological characteristics, including age, serum tumor marker levels, poor differentiation, and the number of tumor lesions.

Recent evidence has highlighted the association between changes in inflammatory biomarkers and the inflammatory immune response within localized tumors (22, 30). They have demonstrated satisfactory objective response rates in clinical settings (13, 31). This might be attributed to the combination therapy exerts synergistic anti-tumor activity and the induced local tumor-related inflammatory immune response. By inhibiting tumor angiogenesis and regulating the tumor microenvironment, it enhances the effectiveness of immunotherapy. Inflammatory factors within the tumor microenvironment exert a pivotal influence in HCC treatment, and inflammatory biomarkers have demonstrated the capacity to predict the therapeutic efficacy and adverse reactions of LPI combination therapy (32). Prior studies have identified PNI and SII as risk indicators for OS (33, 34); our results further demonstrate that these inflammatory biomarkers are also correlated with tumor recurrence and RFS in patients. Nomograms are practical tools for developing graphical prediction models, offering robust predictive and discriminative validity for individual patients and aiding clinical management (35). Compared with traditional staging systems like BCLC and CNLC, the nomogram in this study showed enhanced accuracy in predicting tumor recurrence. This finding highlights its potential as a superior, individualized predictive tool for patients undergoing LPI-based therapies.

This study retrospectively analyzed HCC patients who received LPI-based conversion hepatectomy, developing and validating two nomograms for predicting recurrence probability and RFS. These nomograms assist clinicians in assessing recurrence risk and planning postoperative management. Patients undergoing conversion hepatectomy typically present with more advanced tumor stages at diagnosis and experience higher recurrence rates compared to those undergoing initial radical resection (36). Thus, shortening follow-up intervals for early recurrence detection and timely intervention is crucial. For high-risk patients (e.g., with microvascular invasion, multiple lesions, or poor differentiation), adjuvant therapy may be necessary, while reducing postoperative treatment intensity can prevent overtreatment in low-risk patients.

Several limitations of this study warrant acknowledgement. First, although these nomograms have a strong C-index of 0.84, their development from a single-center cohort highlights the need for multicenter external validation to ensure their broader applicability and clinical effectiveness. Second, the limited cohort size may hinder subgroup analyses. Since LPI has been gradually integrated into HCC conversion therapy in recent years, it will take time to accumulate larger resection cohorts and perform multi-center validations. Thirdly, in clinical practice, 81.3% of patients received TACE and 18.7% HAIC, reflecting patient-specific factors such as tumor burden, vascular invasion, and liver function. However, treatment heterogeneity may introduce confounding factors affecting study results. Future studies should prioritize more homogeneous treatment groups, potentially through refined selection criteria or stratified randomization, to improve research robustness. Lastly, the nomogram is based on easily obtainable clinicopathological factors that may not fully reflect tumor biology. Future work should explore more specific markers (e.g., genetic or immune markers) to boost prediction accuracy. Despite these limitations, our work unequivocally advances the individualized management of HCC patients following conversion hepatectomy.

## Conclusion

This study successfully developed two highly accurate nomogram models to estimate the likelihood of tumor recurrence and RFS among individuals with HCC undergoing conversion hepatectomy based on the LPI regimen. Our research outcomes may help clinicians develop personalized management protocols for individuals undergoing LPI-based hepatectomy. Future efforts should focus on refining the nomogram models and evaluating their real-world applicability.

## Data Availability

The raw data supporting the conclusions of this article will be made available by the authors, without undue reservation.
